# Supplementary material for: Identification of photoperiod-induced specific miRNAs in the adrenal glands of Sunite sheep (Ovis aries)
Source: Front Vet Sci. 2022 Jul 22;9:888207. doi: 10.3389/fvets.2022.888207 (PMC9354845; doi:10.3389/fvets.2022.888207)
Supplement: Supplementary file 1 [file Data_Sheet_1.ZIP › Supplementary file/miRDeep2 file.zip!/miRDeep2 file/Novel_Structure/SPLP42A3/Novel_183.pdf]

Provisional ID	:	NC_019464.2_16045
Score total	:	136.8
Score for star read(s)	:	3.9
Score for read counts	:	129.1
Score for mfe	:	2.2
Score for randfold	:	1.6
Score for cons. seed	:	
Total read count	:	265
Mature read count	:	254
Loop read count	:	0
Star read count	:	11

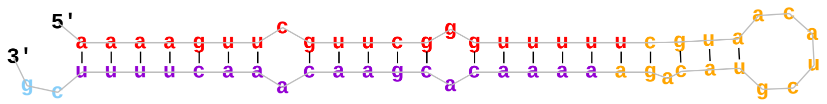

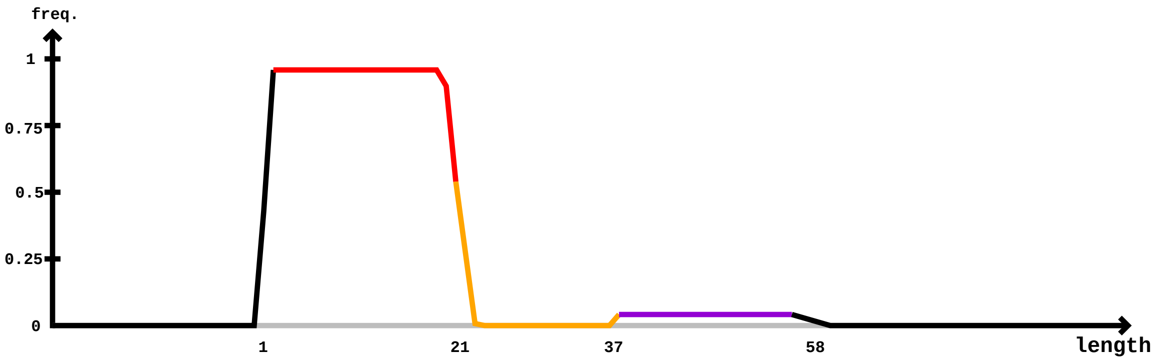

Mature **Star**

```
5'- auuaucauauuggguuggcugaaaaguucguucggguuuuucguaacaucguacagaaaaacacgaacaaacuuuucggccaacccaguauuucauucaucugcguuuuc   -3'    obs
    auuaucauauuggguuggcugaaaaguucguucggguuuuucguaacaucguacagaaaaacacgaacaaacuuuucggccaacccaguauuucauucaucugcguuuuc          exp
    ......((((((((((((((((((((((.(((((.((((((((((......))).))))))).))))).)))))))))))))))))))))).......................   reads    mm    sample
    ....................gaaaaguucguucggguuu.......................................................................   14       0     seq
    ....................gaaaaguucguucggguuuu......................................................................   95       0     seq
    ....................Aaaaaguucguucggguuuuu.....................................................................   5        1     seq
    .....................aaaaguucguucggguuu.......................................................................   2        0     seq
    .....................aaaaguucguucggguuuuu.....................................................................   103      0     seq
    .....................aaaaguucguucggguuuuuc....................................................................   33       0     seq
    .....................aaaaguucguucggguuuuucU...................................................................   2        1     seq
    ........................................................aaaacacgaacaaacuuuu...................................   9        0     seq
    ........................................................aaaacCcgaacaaacuuuuc..................................   2        1     seq
```
